# Retraction Note: Unique RNA signature of different lesion types in the brain white matter in progressive multiple sclerosis

**DOI:** 10.1186/s40478-019-0790-7

**Published:** 2019-08-21

**Authors:** Maria L. Elkjaer, Tobias Frisch, Richard Reynolds, Tim Kacprowski, Mark Burton, Torben A. Kruse, Mads Thomassen, Jan Baumbach, Zsolt Illes

**Affiliations:** 1 Department of Neurology, Odense University Hospital, J.B. Winslowsvej 4, DK-5000 Odense, Denmark; 2 Institute of Clinical Research, BRIDGE, University of Southern Denmark, Odense, Denmark; 3 Institute of Molecular Medicine, University of Southern Denmark, Odense, Denmark; 4 Department of Mathematics and Computer Science, University of Southern Denmark, Odense, Denmark; 5 Division of Brain Science, Imperial College, London, UK; 6 Research Group Computational Systems Medicine, Chair of Experimental Bioinformatics, TUM School of Life Sciences Weihenstephan, Technical University of Munich, Munich, Germany; 7 Department of Clinical Genetics, Odense University Hospital, Odense, Denmark; 8 Chair of Experimental Bioinformatics, TUM School of Life Sciences Weihenstephan, Technical University of Munich, Munich, Germany

**Retraction Note: Acta Neuropathol Commun**

**https://doi.org/10.1186/s40478-019-0709-3**

The authors have retracted this article [1] because a line was omitted from the data sheet; this was due to a bug in the analysis scripts. This resulted in shifting labels, and incorrect label annotation files for 10 out of 100 samples.

The authors have repaired the labels and re-analyzed the data. While many of the conclusions remained unchanged and validated by other methods, some conclusions cannot hold anymore. A detailed description of the major changes is as followed:

1. The conclusion in the original paper was that NAWM is more similar to WM control than to MS lesions (Fig.3). This was based on the low number of significantly differentially expressed genes (DEGs) between NAWM and control WM. Now, the number of DEGs in the NAWM increased significantly compared to control WM: several new genes have been discovered, and the authors also found 16 unique genes in NAWM that are differentially expressed and are not present in any lesion type (see Figure 1 below).
2. The conclusion in the original paper that chronic active lesion was the most distinct lesion type is still valid, as a new Venn diagram (Figure 2 in original article, see Fig. 1 below) now indicates that chronic active lesion has the highest number of unique genes compared to the other lesion types. However, the heatmap presented in Figure 4 that showed 62 genes separating chronic active lesions from the rest of lesion types is now incorrect: the new heatmap consists of 965 genes, where only 24 of those 62 genes in Figure 4 are still present. Further analyses are ongoing.
3. The conclusion in the paper that the authors found three molecular markers that may be specific for the different lesions has also been changed. The gene of CD26 is still significantly upregulated in NAWM and expressed by microglia. (ii) However, although CHI3L1 is present in chronic active lesions by astrocytes in the rim, verified with immunohistochemistry (IHC) and RNAscope, its gene expression is not significant. (iii) The authors also found TGFbeta-R2 in a repair specific network (Fig.6) and verified its presence in astrocytes in remyelinating lesions by RNAscope and IHC. However, TGFBR2 is significantly upregulated in other lesion types as well.

**Fig. 1:**
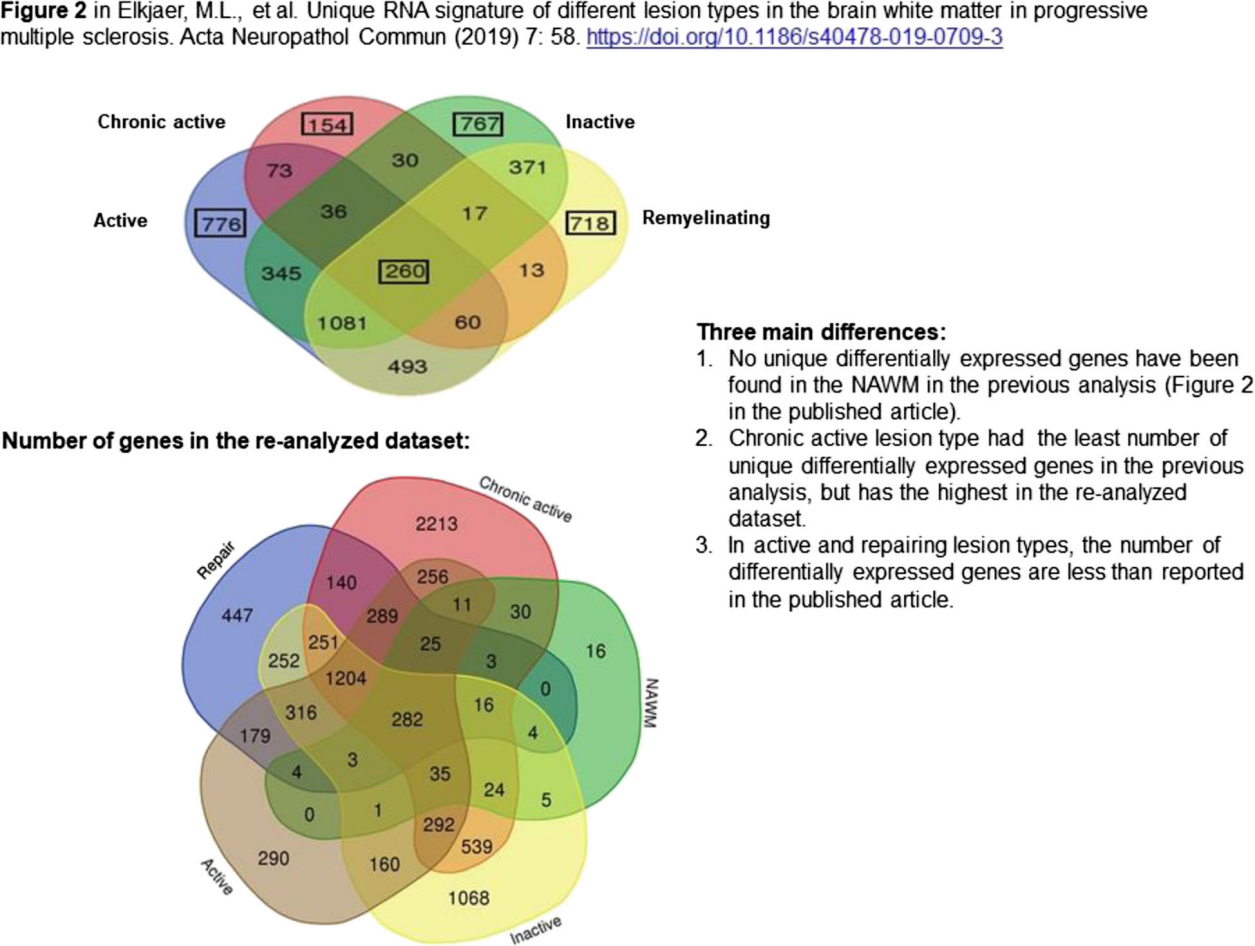
Updated Venn diagram

**All authors agree to this retraction.** The authors have been offered to submit a revised manuscript for further peer review.
